# Development and Function of CD94-Deficient Natural Killer Cells

**DOI:** 10.1371/journal.pone.0015184

**Published:** 2010-12-03

**Authors:** Mark T. Orr, Jun Wu, Min Fang, Luis J. Sigal, Pieter Spee, Thomas Egebjerg, Erik Dissen, Sigbjørn Fossum, Joseph H. Phillips, Lewis L. Lanier

**Affiliations:** 1 Department of Microbiology and Immunology and the Cancer Research Institute, University of California San Francisco, San Francisco, California, United States of America; 2 DNAX Research Institute, Palo Alto, California, United States of America; 3 Immune Cell Development and Host Defense Program, Fox Chase Cancer Center, Philadelphia, Pennsylvania, United States of America; 4 Department of NK Cell Biology, Novo Nordisk A/S, Måløv, Denmark; 5 Department of Anatomy, Institute of Basic Medical Sciences, University of Oslo, Oslo, Norway; Statens Serum Institute, Denmark

## Abstract

The CD94 transmembrane-anchored glycoprotein forms disulfide-bonded heterodimers with the NKG2A subunit to form an inhibitory receptor or with the NKG2C or NKG2E subunits to assemble a receptor complex with activating DAP12 signaling proteins. CD94 receptors expressed on human and mouse NK cells and T cells have been proposed to be important in NK cell tolerance to self, play an important role in NK cell development, and contribute to NK cell-mediated immunity to certain infections including human cytomegalovirus. We generated a gene-targeted CD94-deficient mouse to understand the role of CD94 receptors in NK cell biology. CD94-deficient NK cells develop normally and efficiently kill NK cell-susceptible targets. Lack of these CD94 receptors does not alter control of mouse cytomegalovirus, lymphocytic choriomeningitis virus, vaccinia virus, or *Listeria monocytogenes*. Thus, the expression of CD94 and its associated NKG2A, NKG2C, and NKG2E subunits is dispensable for NK cell development, education, and many NK cell functions.

## Introduction

Natural killer (NK) cells play an important role in the innate immune response to infections and malignancies by directly killing pathogen-infected or transformed cells and producing cytokines and chemokines that help shape the immune response [1]. NK cell activation is controlled by a number of activating and inhibitory receptors [2]. Activating NK receptors recognize a variety of stress-induced molecules such as the human MICA and MICB molecules, human ULBP1-6 proteins, mouse Rae-1, H60, and MULT1 family members, all of which are recognized by the activating NKG2D receptor [3]. Some activating NK receptors directly recognize pathogen-encoded ligands. For example, the m157 glycoprotein expressed by MCMV is recognized by the activating Ly49H receptor and NKp46 interacts with influenza hemagglutinin [4], [5], [6]. NK cells are prevented from attacking normal self cells by inhibitory receptors that are reactive to MHC class I [7].

In mice, the inhibitory members of the Ly49 C-type lectin-like receptor family of NK cell receptors are the primary MHC class I receptors [8]. Humans do not express Ly49 receptors; instead, human NK cells express the structurally unrelated inhibitory killer cell immunoglobulin-like receptors (KIRs) that bind to MHC class I. Additionally, humans and mice both express members of the NKG2 family receptors that form obligate disulfide-bonded heterodimers with CD94. When expressed at physiological levels, the human NKG2 proteins cannot be stably expressed on the cell surface without CD94 [9], [10], [11]. CD94-NKG2 receptors bind non-classical MHC class Ib molecules, HLA-E in humans and Qa-1 in mice [12], [13], [14]. HLA-E and Qa-1 both present conserved peptides derived from the leader segments of classical MHC class I molecules [12], [15]. Both human and murine NKG2 families consist of three members that share a high degree of similarity in their extracellular domains, NKG2A, NKG2C, and NKG2E [13], [16], [17], [18]. NKG2D is an unrelated receptor that does not pair with CD94 and has low sequence homology with NKG2A, NKG2C, and NKG2E [19].

In adult mice, CD94 is expressed on splenic NK cells and NKT cells and a small proportion of γδ T cells and CD8^+^ T cells, primarily CD44^hi^ memory cells. In mouse NK cells, NKG2A is the predominant NKG2 family member with NKG2A mRNA being more prevalent than NKG2C and NKG2E transcripts [17]. NKG2A is the only NKG2 family member expressed in mouse T cells [17]. The cytoplasmic domain of NKG2A contains one canonical immunoreceptor tyrosine-based inhibitory motif (ITIM) in mice and two ITIMs in human [9], [13], [20], [21]. Accordingly, triggering of CD94-NKG2A suppresses NK cell functions. NKG2C and NKG2E lack known intracellular signaling domains, but instead contain a charged residue within their transmembrane domains that facilitate binding to the DAP12 signaling adapter molecule, which contains an immunoreceptor tyrosine based activating motif (ITAM) [16]. Triggering of CD94-NKG2C leads to phosphorylation of the DAP12 ITAM and signaling via Syk and ZAP-70, resulting in NK cell activation [20], [21]. As with other paired inhibitory and activating ligands, the affinity for HLA-E is 10-fold higher for the inhibitory human CD94-NKG2A than the activating CD94-NKG2C receptor [22], [23]. The affinities of the mouse CD94-NKG2 receptors for Qa-1 have not been reported. To better understand the role of CD94-NKG2 receptors in NK cell development and function we disrupted the gene encoding CD94 (*Klrd1*) in the genome of 129/SvJ strain ES cells, generated mice lacking CD94, and backcrossed this CD94-deficient mouse onto the C57BL/6 background. Additionally, we generated a CD94 transgenic mouse that was crossed with the CD94-deficient mice to restore the expression of CD94-NKG2A, CD94-NKG2C, and CD94-NKG2E receptors in the *Klrd1^−/−^* mice to evaluate the role of these receptors in NK cell development and function.

## Results

### Generation and phenotype of CD94-deficient mice

**Figure 1:**
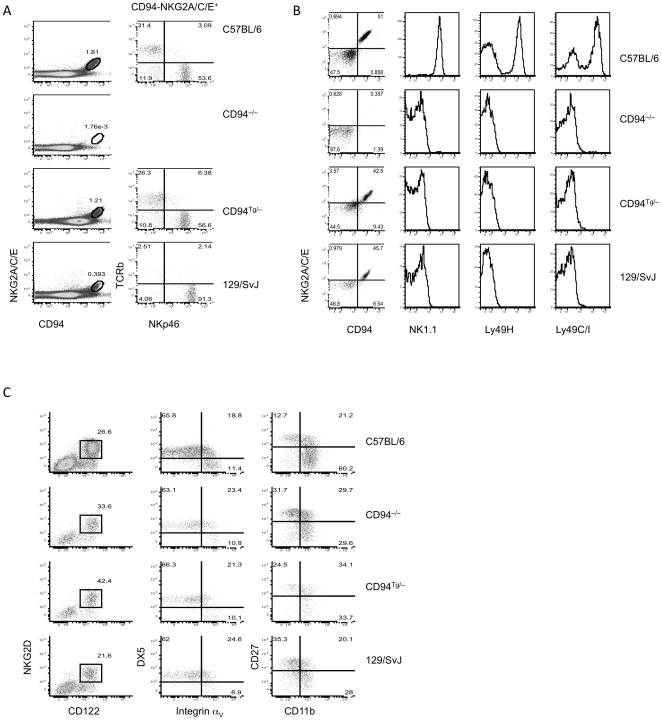
Splenic CD94-deficient and CD94^Tg/–^ NK cells are phenotypically normal. (A) Splenocytes from B6, CD94-deficient, CD94^Tg/–^, and 129/SvJ mice were analyzed for CD94-NKG2 expression. CD94-NKG2^+^ cells were analyzed for NKp46 and TCRβ expression as shown in the second column. (B) NK cells (NKp46^+^ TCRβ^–^) were analyzed for expression of CD94, NKG2A/C/E, NK1.1, Ly49C/I, and Ly49H. (C) CD19^–^ bone marrow cells were analyzed for NK cell precursors (NKG2D^+^ CD122^+^) and the developmental markers DX5, α_V_, CD27, and CD11b. Data are representative of three to five experiments each.

To understand the contribution of CD94 receptors to NK cell and T cell functions we generated a CD94-deficient mouse by targeted disruption of exons 3 and 4 of *Klrd1* in 129/SvJ ES cells and backcrossing the null gene into the genome of C57BL/6 mice. Splenocytes from CD94-deficient mice failed to express the CD94-NKG2A, CD94-NKG2C, or CD94-NKG2E receptors (Fig. 1A). Transgenic expression of CD94 in these CD94-deficient mice (designated CD94^Tg/–^ mice) restored expression of CD94-NKG2A, CD94-NKG2C, and CD94-NKG2E (Fig. 1A). Although a MHC class I promoter with an Igµ enhancer drove the CD94 transgene [24], CD94 was expressed at the highest levels on NKp46^+^ NK cells (Fig. 1A). Similar to B6 mice, the majority of the remaining CD94-NKG2-expressing cells from CD94^Tg/–^ mice were NKT cells and T cells (Fig 1A). CD94^Tg/–^ splenocytes did show an increased intensity of CD94 staining as determined by mean fluorescence intensity (MFI) on the NKG2A/C/E^–^ cells as compared to B6, CD94-deficient, and 129/SvJ splenocytes (MFI 4433, 625, 492, and 1013, respectively) (Fig. 1A). NKG2A/C/E expression levels, as determined by staining with an antibody that crossreacts with NKG2A, NKG2C, and NKG2E, were consistently lower in the CD94^Tg/–^ splenocytes (MFI 147) when compared with B6 splenocytes (MFI 213); however, the MFI of NKG2A/C/E was similar between the CD94^Tg/–^ and 129/SvJ splenocytes (MFI 135), suggesting allelic differences between the B6 and 129/SvJ genes encoding NKG2 receptors might determine the surface density of CD94-NKG2A/C/E (Fig. 1A). Transgenic expression of CD94 restored CD94-NKG2 expression to approximately half of the NK cells in these mice, similar to wildtype mice (Fig. 1B). This suggests that expression of NKG2A, NKG2C, or NKG2E, not CD94, might be the limiting factor in CD94-NKG2 surface expression.

*Klrd1* is located between the Ly49 gene cluster and the NKR-P1 gene cluster in the NK complex (NKC) found on chromosome 6 [25], [26]. B6 and 129/SvJ mice carry different loci and alleles of this genomic cluster, with NK cells from B6 mice but not 129/SvJ mice expressing Ly49C, Ly49H, and NKR-P1C (NK1.1) [27]. NK cells from CD94-deficient mice did not express any of these receptors, indicating that despite being backcrossed to B6 for 9 generations they retained the NKC of 129/SvJ strain mice, at least spanning the regions containing the NKR-P1 and Ly49 loci (Fig. 1B).

CD19^–^ CD122^+^ NKG2D^+^ NK cell precursors undergo an orderly development in the bone marrow that can be distinguished based on the expression of the integrins α_V_ (CD51) and DX5 (CD49b) [28]. α_V_ is expressed first by NK precursors, followed by co-expression of DX5, and finally loss of α_V_ expression. CD94-NKG2 receptors are initially expressed by α_V_^+^, DX5^–^ immature NK cells [28]. CD27 and CD11b can also delineate NK cell maturation stages. CD27^+^CD11b^lo^ NK cells are the most immature with CD11b expression increasing as NK cells mature, and CD27 is lost on the most mature NK cells [29]. NK cell precursors from CD94-deficient, CD94^Tg/–^, and 129/SvJ mice contained similar frequencies of each these developmental stages, indicating that expression of CD94-NKG2 is not necessary for normal NK development (Fig. 1C). The frequency of CD11b^hi^ CD27^–^ mature NK cells was somewhat higher in the B6 mice than any of the other strains, suggesting that a factor other than CD94-NKG2 expression may differentially regulate NK cell development in B6 vs. 129/SvJ mice. Frequencies and absolute numbers of splenic NK cells were similar among B6, CD94-deficient, CD94^Tg/–^, and 129/SvJ mice (data not shown).

### NK cell functions are not altered in CD94-deficient mice

**Figure 2:**
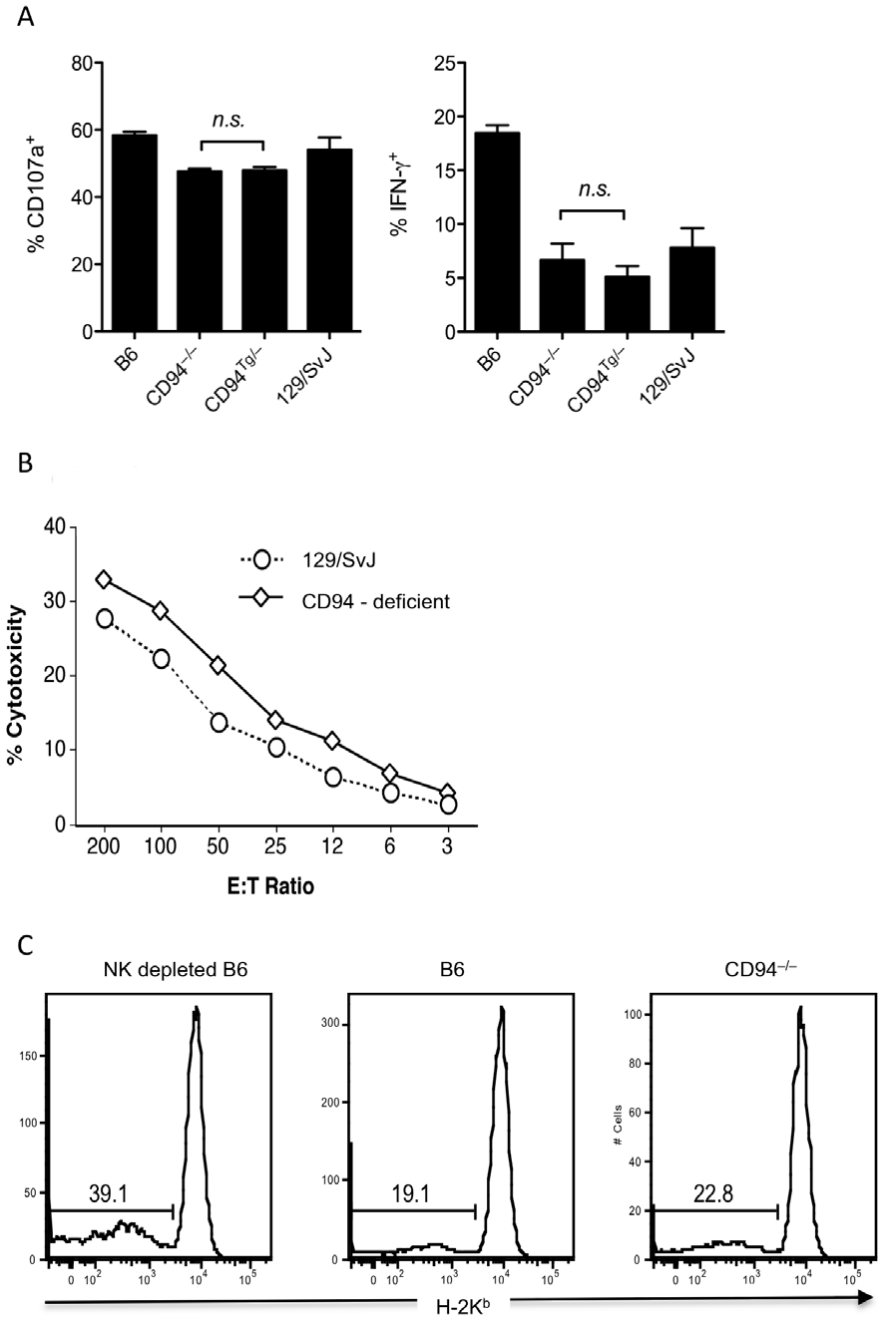
CD94-deficient NK cells are educated and efficiently kill YAC-1 targets and MHC I-deficient splenocytes. (A) Splenocytes from B6, CD94-deficient, CD94^Tg/–^, and 129/SvJ mice were assayed for degranulation as measured by surface staining for CD107a and intracellular IFN-γ production upon stimulated with plate-bound anti-NKp46 mAb. (B) Splenocytes from CD94-deficient mice and wildtype 129/SvJ mice primed with poly I:C *in vivo* were assayed for NK cell-mediated cytotoxicity against the YAC-1 target cell. (C) CD94-deficient mice and B6 mice either depleted of NK cells with anti-NK1.1 mAb or treated with PBS received a mixture of CFSE-labeled wildtype and *B2m^−/−^* B6 splenocytes. Twenty-four hours later the frequency or CFSE-labeled donor cells in the spleen that were *B2m^−/−^*was determined by expression of H-2K^b^. Data are representative of three to five experiments each.

Expression of inhibitory receptors for self-MHC class Ia and Ib molecules, including the Ly49 receptors and CD94-NKG2A, enhances NK cell responsiveness to activation by crosslinking of activating receptors [30], [31], [32], [33]. To determine whether CD94-deficiency affects NK cell education, we stimulated B6, CD94-deficient, CD94^Tg/–^, and 129/SvJ NK cells by crosslinking the activating NKp46 receptor. CD94-deficient, CD94^Tg/–^, and 129/SvJ NK cells all produced IFN-γ and degranulated at similar frequencies as measured by CD107a surface staining (Fig. 2A). This indicates that CD94 is not required for NK cell education in NK cells carrying the 129/SvJ NK complex. CD94-deficient, CD94^Tg/–^, and 129/SvJ NK cells all produced IFN-γ and degranulated less frequently than B6 NK cells, suggesting that an unknown factor within the NK complex might control the degree to which B6 and 129/SvJ NK cells are educated (Fig. 2A)

NK cells lyse target cells expressing ligands for a variety of NK receptors, including the NKG2D receptor necessary for recognition of the prototypic NK-sensitive YAC-1 tumor cell line. NKG2D expression was similar between B6 and CD94-deficient NK cells (data not shown). Accordingly, NK cells from both B6 and CD94-deficient mice killed YAC-1 targets with similar efficacy, indicating that CD94-deficiency did not affect NK cell lytic capacity against this target cell (Fig. 2B).

NK cells are prevented from killing healthy bone marrow cells and splenocytes by inhibitory receptors on NK cells that engage MHC class I on these target cells. Bone marrow cells and splenocytes from animals deficient in surface expression of MHC class I due to deletion of the *B2m*, *TAP1* and *TAP2*, or *H-2K* ^b^ and *H-2D^b^* genes are efficiently eliminated by NK cells *in vivo* [34], [35]. To test whether CD94-NKG2 receptors were important in “missing-self” recognition we transferred a 1∶1 mixture of wildtype and *B2m^−/−^* splenocytes into B6 mice depleted of NK cells or treated with PBS or into CD94-deficient mice. Twenty-four hours later both B6 and CD94-deficient mice efficiently eliminated the *B2m^−/−^* splenocytes compared to B6 mice depleted of NK cells (Fig. 2C).

### CD94-NKG2 receptors do not contribute to NK cell control of MCMV

**Figure 3:**
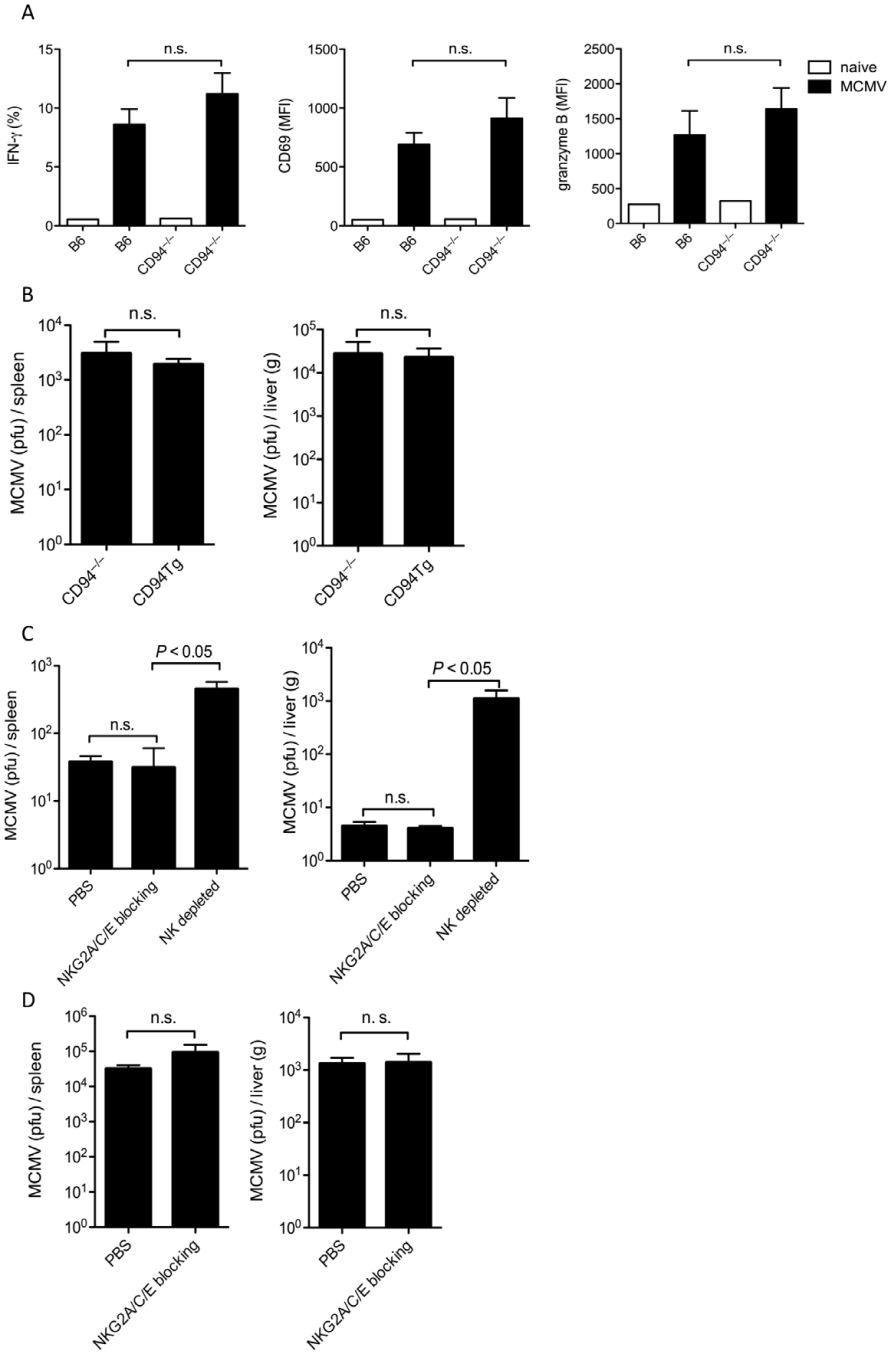
CD94 is not necessary for control of MCMV. (A) Two days after infection with MCMV splenic NK cells from B6 and CD94-deficient mice were analyzed for expression of CD69 and intracellular IFN-γ and granzyme B. (B) CD94-deficient and CD94^Tg/–^ mice, (C) B6 mice treated with the non-depleting, blocking anti-NKG2A/C/E chimeric rat-mouse monoclonal antibody 20D5HCmIgG1-Q, anti-NK1.1 depleting antibody or PBS (D), or BALB/c mice receiving the 20D5HCmIgG1-Q antibody or PBS were infected with 5×10^4^ pfu MCMV and then analyzed for viral titers three days later. Graphs represent the average ± s.e.m. of four or five animals per group. Not statistically significant (n.s.).

After MCMV infection all NK cells are activated, likely due to the inflammatory cytokine milieu induced by the infection [36]. Despite not expressing the MCMV specific Ly49H receptor (Fig. 1B), CD94-deficient NK cells upregulated expression of CD69, granzyme B, and IFN-γ to a similar extent as B6 NK cells (Fig 3A). CD94-deficient and CD94^Tg/–^ mice were equally susceptible to MCMV infection, suggesting that in the absence of the Ly49H activating receptor CD94-NKG2 does not play an important role in the control of MCMV (Fig. 3B). CD94-deficient and CD94^Tg/–^ NK cells express the 129/SvJ allele of the Ly49I inhibitory receptor, which binds to MCMV m157 [6]. This interaction may mask any effect CD94-NKG2 receptors could have on control of MCMV. To determine whether CD94-NKG2 receptors affect NK cell control in mice that express the MCMV-specific Ly49H activating receptor we treated B6 mice either with PBS or a non-depleting, blocking antibody that recognizes NKG2A, NKG2C, and NKG2E. Despite saturating the CD94-NKG2 receptors with the neutralizing antibody (data not shown), this treatment had no effect on NK cell control of MCMV infection in these mice (Fig. 3C). Blocking NKG2A, NKG2C, and NKG2E also did not affect control of MCMV in the susceptible BALB/c mice that do not express Ly49H or Ly49I (Fig. 3D). Thus CD94-NKG2 receptors do not affect the NK cell response to infection with the Smith strain of MCMV.

### CD94-deficient mice control LCMV, vaccinia virus, and Listeria infection as efficiently as B6 mice

**Figure 4:**
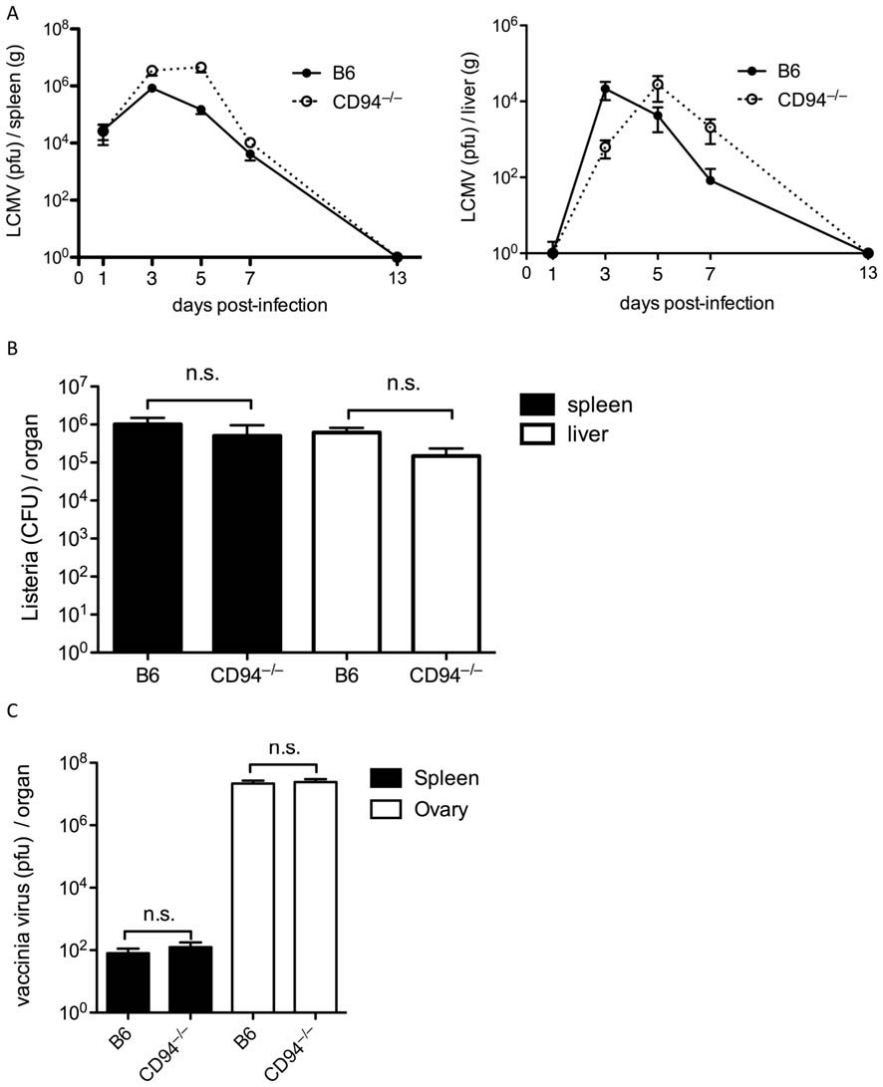
CD94 is not necessary for clearance of LCMV, vaccinia virus or Listeria infection. (A) B6 and CD94-deficient mice were infected with 2×10^5^ pfu of LCMV (Armstrong strain). Spleens and livers were analyzed for LCMV titers on the indicated days. (B) B6 and CD94-deficient mice were challenged with 1×10^5^ cfu *Listeria monocytogenes*. Spleens and livers were analyzed for bacterial burdens three days later. (C) B6 and CD94-deficient mice were challenged with 5×10^6^ pfu vaccinia virus. Spleens and ovaries were analyzed for viral titers seven days later. Graphs represent the average ± s.e.m. of three to five animals per group per time point.

To determine whether CD94-NKG2 receptors contributed to the clearance of other infections we infected CD94-deficient and B6 mice with LCMV, vaccinia virus or *Listeria monocytogenes*. LCMV clearance was slightly delayed in the CD94-deficient mice with viral loads in the spleens and livers of B6 mice peaking at day 3 compared to day 5 in the CD94-deficient mice (Fig 4A). However, differences in viral titers were not statistically significant. Despite this delay in peak viral load, CD94-deficient mice cleared the infection by day 13 suggesting that CD94-NKG2 does not play a prominent role in control of LCMV infection. The role of NK cells in the control of *Listeria monocytogenes* infection is controversial, with some studies suggesting that NK cells provide protection against Listeria and others that NK cells promote bacterial growth [37]. Three days after challenge with Listeria, the bacterial burden in the spleens and liver was similar between CD94-deficient and B6 mice (Fig 4B). Similarly, control of vaccinia virus replication was comparable in CD94-deficient and B6 mice (Fig. 4C).

## Discussion

We have generated a mouse with a targeted deletion of the *Klrd1* gene encoding CD94. CD94-NKG2A is the first inhibitory receptor for MHC class I that is expressed on developing NK cells and is the predominant inhibitory receptor expressed on fetal NK cells [38]. Previous work proposed that CD94-NKG2A might be necessary for self-tolerance in these developing NK cells [39], [40]. We find, however, that deletion of *Klrd1* neither affected the maturation or development of NK cells in the bone marrow, nor did it influence the numbers or frequency of mature splenic NK cells, suggesting other mechanisms may ensure NK self tolerance in the absence of CD94-NKG2A. Similarly, DBA/2J mice have a spontaneously arising mutation in the *Klrd1* gene that prevents expression of CD94, yet NK cells develop normally in these mice and do not cause overt autoimmunity [24]. Compensatory mechanisms mediated by other inhibitory receptors might facilitate NK cell education in these CD94-deficient mice [31], [32]. Loss of CD94-NKG2A in the CD94-deficient mice might be predicted to render the developing NK cells hypo-responsive; however, CD94-deficient splenic NK cells produced IFN-γ and degranulated at similar frequencies to CD94^Tg/–^ and 129/SvJ NK cells. Additionally, mature CD94-deficient NK cells were competent to kill YAC-1 cells *in vitro* and *B2m^−/−^* splenocytes in vivo, indicating that these cells were not impaired in their lytic functions. Depletion of CD94-NKG2A^+^ NK cells was previously shown to impair rejection of *B2m^−/−^* splenocytes *in vivo* by B6 NK cells [31]. Sorted CD94^–^ NK cells from B6 mice were also reported to be less cytolytic against YAC-1 targets than their CD94^+^ counterparts [41]. These discrepancies between our findings and prior reports could be accounted for by the differential Ly49 repertoire expressed by CD94-deficient NK cells compared to B6 NK cells. Specifically, four of the inhibitory Ly49 receptors in the 129/SvJ NK receptor haplotype expressed by the CD94-deficient NK cells are reactive to H-2^b^ (Ly49G2, I, O, and V), whereas only two of the Ly49 receptors in B6 bind H-2^b^ (Ly49C and I) [8], [42], [43]. This increased repertoire of H-2^b^-reactive inhibitory receptors contributed by the 129/SvJ NKC might render CD94-NKG2A redundant in the education of NK cells expressing the 129/SvJ NKC. The lack of monoclonal antibodies specific for 129/SvJ Ly49 receptors prevents formal testing of this hypothesis. Additionally, in B6 mice CD94^–^ NK cells may represent a less mature NK cell population that CD94^+^ NK cells, thus complicating comparisons between CD94^–^NK cells in wildtype B6 mice and CD94-deficient NK cells bearing the 129/SvJ NKC.

Despite ubiquitous expression of the CD94 transgene in CD94-deficient mice, surface expression of CD94-NKG2 was restored in only half of the NK cells and a small fraction of T cells and NKT cells (similar to expression of CD94-NKG2 in wildtype mice), indicating that transcription of NKG2A, NKG2C, and NKG2E is likely the rate-limiting factor in CD94 surface expression. Similarly, the expression of the CD94 transgene in DBA2/J mice, which lack CD94 expression, restored CD94-NKG2 expression in only a subset of NK cells [24]. The low level of CD94 surface expression in all cells from the CD94^Tg/–^ mice is likely due to the CD94 transgene being expressed at a low level as a homodimer on all cells. Crystal structures of CD94-NKG2A and CD94-NKG2C show that both CD94 and the NKG2 receptors make contact with HLA-E, suggesting that CD94 homodimers might bind Qa-1 [44], [45], [46]. The intracellular domain of CD94 consists of only 10 amino acids and lacks any known signaling domain; thus, the CD94 homodimers in the CD94^Tg/–^ mice are not likely to be functional [47].

In humans, co-culture with HCMV-infected fibroblasts led to a selective expansion of human CD94-NKG2C^+^ NK cells, and CD94-NKG2C expression correlated with positive serology for HCMV, suggesting that CD94-NKG2C may directly recognize HCMV-infected cells [48], [49], [50]. MCMV is often used to model immunity to HCMV. B6 mice express the activating Ly49H receptor, which binds to the MCMV m157 glycoprotein expressed on the surface of infected cells and accounts for the NK cell-mediated resistance to MCMV in this strain [5], [6]. Blocking NKG2A/C/E receptors in B6 mice did not affect control of MCMV infection. This may be due to the strong activation via Ly49H, overriding any effect CD94-NKG2A/C/E receptors might have had on NK cell control of MCMV. Unlike B6 mice, BALB/c mice do not express Ly49H and are susceptible to MCMV. Blocking NKG2A/C/E in BALB/c mice also did not affect control of MCMV, suggesting that Ly49H did not mask a role for CD94-NKG2A/C/E in control of MCMV. This is in agreement with a prior report demonstrating that viral clearance from the submandibular glands is unaltered in CD94-deficient DBA/2J mice (which share the BALB/c Ly49 genes) compared to DBA/2J mice expressing a CD94 transgene, despite increased expression of Qa-1 in the submandibular glands after viral infection [51]. The 129/SvJ allele of Ly49I expressed in CD94-deficient and CD94^Tg/–^ mice binds the MCMV m157 glycoprotein and might prevent NK cell responses to MCMV-infected cells [6]. Lack of CD94 did not enhance control of MCMV. Thus, despite potentially playing an important role in the NK cell response to HCMV, in mice CD94-NKG2 receptors do not appear to be essential for the NK cell response to MCMV in the mouse strains tested, possibly due to redundancies in the NK response to MCMV. This may be due to different ligands for CD94-NKG2C induced by HCMV or different peptides being presented by HLA-E during infection that alter CD94-NKG2C activity. Additionally CD94-deficienct mice did not alter resistance to LCMV, vaccinia virus, or *Listeria monocytogenes*, but might be important for control of other pathogens. For example, CD94-NKG2A expression on polyoma virus-specific CD8^+^ T cells limits their cytolytic capacity when engaging Qa-1-expressing target cells [52]. Thus, further studies are warranted to uncover the physiological role of CD94-NKG2 receptors in NK cell-mediated host defense.

## Materials and Methods

### Mice

C57BL/6 (B6) and 129/SvJ mice purchased from the National Cancer Institute and Jackson Labs respectively. All mice were maintained in the specific pathogen-free animal facility of the University of California San Francisco or Fox Chase Cancer Center. The Fox Chase Cancer Center Institutional Animal Care (A3285-01) and Use Committee and the UCSF Institutional Animal Care and Use Committee (AN080996-02E) approved animal protocols.

### Generation of CD94-deficient and CD94^Tg^ mice

A fragment of 129-strain mouse genomic DNA was isolated and the *Klrd1* gene was mapped and sequenced. Like human *KLRD1* [53], the mouse gene is encoded by 6 exons. The neomycin-resistance gene, flanked by lox sites, was used to disrupt *Klrd1*, resulting in the partial elimination of exon 3 and complete removal of exon 4. The deleted genetic segments contain the extracellular region with the cysteine residue required for dimerization with NKG2A, NKG2C, and NKG2E and removed a substantial amount of the C-type lectin-like domain. Homologous recombination in 129 ES cells was confirmed by Southern blot analysis and the neomycin-resistance gene was deleted by transient transfection of the cells with the Cre recombinase to avoid effects on expression of neighboring genes, sometimes caused by the neomycin-resistance gene cassette. Chimeric mice were produced and germline transmission was achieved. Mice were backcrossed to C57BL/6 for 9 generations. A CD94 transgene driven by the class I promoter/Igµ enhancer [24] was injected into CD94-deficient blastocysts to generate CD94^Tg/–^ mice.

### Antibody staining

Splenocytes were stained with anti-NKp46 (29A1.4), anti-Ly49H (3D10), anti-Ly49C/I (5E6), anti-CD94 (18d3), anti-NKG2A/C/E (20D5), anti-NKG2D (CX5), anti-NK1.1 (PK136), anti-TCRβ (H57-597), anti-CD45.1 (A20), anti-CD45.2 (104), anti-CD49b (DX5), anti-integrin α_V_ (RMV-7), anti-CD11b (M1/70), anti-CD27 (LG3.A10), anti-H-2K^b^ (AF6-88.5), anti-CD69 (H1.2F3), anti-granzyme B (GB11), anti-CD107a (1D4B) and anti-IFN-γ (XMG1.2). Anti-NKp46, anti-Ly49H, anti-CD45.1, anti-NKG2D, anti-NKG2A/C/E, and anti-CD94 were purchased from eBioscience. Anti-NK1.1, anti-CD45.2, anti-H-2K^b^, anti-TCRβ, and anti-CD11b were purchased from BioLegend. Anti-Ly49C/I, anti-granzyme B, anti-CD107a, anti-CD69, anti-IFN-γ, anti-integrin α_V_ and anti-CD27 were purchased from BD Biosciences.

### Antibody stimulation of NK cells

IFN-γ production and degranulation were determined by incubating 5×10^5^ splenocytes on plates coated with 10 µg/mL of anti-NKp46 or an isotype-matched control mAb for 5 hrs in the presence of brefeldin A and anti-CD107a. Cells were surface stained for TCRβ and DX5, and for intracellular IFN-γ by using the Intracellular Staining Kit from BD Biosciences.

### NK cell-mediated cytotoxicity assay

CD94-deficient mice and wildtype 129/SvJ strain mice were injected i.p. with 200 µg poly I:C (Sigma) in sterile PBS. After 24 hrs, splenocytes were harvested and analyzed for cytotoxicity in a 4-hr ^51^Cr-release assay against YAC-1 target cells.

### Rejection of *B2m^−/−^* splenocytes

Wildtype and *B2m^−/−^* B6 splenocytes were mixed 1∶1, labeled for ten minutes with 0.5 µM CFSE in PBS, and washed twice in PBS. Ten million labeled splenocytes were transferred i.v. into naïve recipient mice, either untreated or depleted of NK cells by i.p. administration of 200 µg of anti-NK1.1 (PK136) 24 hrs prior to transfer. Twenty-four hours after transfer splenocytes from recipients were analyzed by flow cytometry.

### MCMV infections

Six to ten week-old mice were infected i.p. with 5×10^4^ pfu MCMV (Smith strain). Two days later splenocytes were stained for NK cell activation. For measurement of MCMV viral titers, spleens, salivary glands and livers were collected, homogenized, plated on M2-10B4 cells (American Type Culture Collection) in RPMI-1640 medium without FCS and incubated for 2 h at 37°C. RPMI-1640 medium with 10% (vol/vol) FCS and 0.75% (wt/vol) carboxymethyl cellulose was added and samples were incubated for 7–10 days. Plaques were visualized by staining with crystal violet dye.

### Generation of an NKG2A/C/E blocking antibody

The 20D5 monoclonal antibody reacts with NKG2A, NKG2C, and NKG2E and blocks the binding of these receptors to Qa-1^b^ [17]. 20D5 is a rat IgG2a antibody that depletes NK cells when injected *in vivo*. To generate a non-depleting antibody that blocks NKG2A, NKG2C, and NKG2E, a chimeric 20D5 heavy chain cDNA sequence was made consisting of rat 20D5 VH-CH1-hinge cDNA and mouse IgG1 CH2-CH3 cDNA by two rounds of PCR-generating overlapping PCR products. In order to diminish Fc effector functions of 20D5HCmIgG1, a 20D5HCmIgG1 cDNA sequence variant was made designated 20D5HCmIgG1-Q encoding 20D5mIgG1 without a conserved N-glycosylation consensus site. The N297Q point mutation was made by using site-directed mutagenesis (Quickchange site-directed mutagenesis II kit, Stratagene). The rat-mouse 20D5HCmIgG1-Q chimeric cDNA sequences were inserted in the pEE14.4 expression vector provided by Lonza Biologics. The resulting pEE14.4-20D5mIgG1-Q expression plasmid, together with the rat κ light chain isolated from the 20D5 hybridoma, was transfected into CHOK1SV cells (Lonza Biologics) and stable 20D5mIgG1-Q expressing CHOK1SV clones were selected based on MSX resistance. The 20D5mIgG1-Q antibody was purified from cell culture supernatants using protein-A based methods.

### LCMV infections

Six to ten week-old mice were infected i.p. with 2×10^5^ pfu LCMV (Armstrong strain). On days 1, 3, 5, 7, and 13 post-infection spleens and livers were harvested in DME with 2% FCS (v/v). Tissues were homogenized, plated on Vero cells (ATCC), and overlaid with 199 media containing 0.5% agarose (w/v) and 10% FCS (v/v). Four days later plaques were visualized by staining with neutral red.

### Listeria infections

Six to ten week-old mice were infected i.p. with 10^5^ cfu log phase *Listeria monocytogenes* grown in tryptic soy broth. Three days later spleens and livers were harvested in PBS with 0.1% (v/v) NP-40, homogenized, and plated on BHI plates.

### Vaccinia infections

Vaccinia virus (WR strain) was inoculated via the i.p. route with 500 µl PBS containing 5×10^6^ pfu virus. Following infections, mice were observed daily for signs of disease (lethargy, ruffled hair, weight loss, skin rash, eye secretions) and imminent death (unresponsiveness to touch, lack of voluntary movements). For the determination of virus titers on the indicated days post-infection, the ovaries were removed and homogenized in medium using a Tissue Lyser (Qiagen), and the spleens were made into a single cell suspension between two frosted slides and resuspended in 10 ml complete RPMI-1640 medium. One ml of the cell suspensions was frozen and thawed three times and viral titers determined by plaque assays on confluent BSC-1 cells using 10-fold serial dilutions of the stocks in 0.5 ml RPMI-2.5 in six-well plates (2 wells/dilution) for 1 h. Two ml fresh RPMI-2.5 was added and the cells incubated at 37°C for 2–3 days. The media was aspirated and the cells fixed and stained for 10 min with 0.1% crystal violet in 20% ethanol. The fix/stain solution was subsequently aspirated, the cells air-dried, the plaques counted, and the pfu/ml in stocks were calculated accordingly.

### Statistical analysis

Statistical differences in viral and bacterial titers were determined by the unpaired, two-tailed Mann-Whitney test. Statistical differences in IFN-γ and CD107 expression were determined by the unpaired, two-tailed Student's t test. Statistics were determined with Prism software (GraphPad Software, Inc.).
